# Q&A: insulin secretion and type 2 diabetes: why do β-cells fail?

**DOI:** 10.1186/s12915-015-0140-6

**Published:** 2015-05-16

**Authors:** James Cantley, Frances M. Ashcroft

**Affiliations:** Department of Physiology, Anatomy & Genetics, University of Oxford, Parks Road, Oxford, OX1 3PT UK

## What is type 2 diabetes?

Diabetes mellitus is a term that covers a multitude of problems with many etiologies, unified by one common feature: the pathological elevation of blood glucose. Sustained hyperglycemia leads to tissue damage in susceptible organs and eventually results in secondary complications including retinopathy, nephropathy, peripheral neuropathy, cardiovascular disease and stroke [1-3]. Diabetes currently affects 387 million people worldwide, and this number is predicted to increase to 592 million by 2035 [4]. The dramatic rise in the disease in recent years not only causes individual misery, but also places an enormous and increasing burden on healthcare systems and the global economy [5,6]. Indeed, many countries spend as much as 10 % of their healthcare budget on treating diabetes and its complications.

**Fig. 1:**
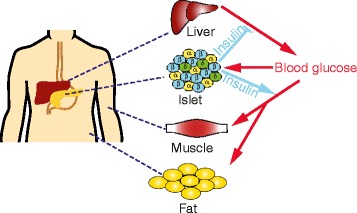
Glucose homeostasis. A rise in blood glucose triggers insulin secretion from β-cells (blue) within the pancreatic islets. Insulin lowers blood glucose by acting on target tissues, suppressing glucose output from the liver and stimulating glucose uptake into muscle and fat. α-cells (yellow) are the glucagon-secreting cells of the pancreas; δ-cells (green) secrete somatostatin

Type 2 diabetes (T2D) is the most common form of the disease, accounting for approximately 90 % of cases [6]. It has a strong genetic component that is amplified by factors such as age, obesity, diet, physical activity and pregnancy. T2D is characterized by insufficient secretion of insulin from the β-cells of the pancreatic islets, coupled with impaired insulin action in target tissues such as muscle, liver and fat (a condition termed insulin resistance). Hyperglycemia results when insulin secretion is unable to compensate for insulin resistance [7]. Insulin resistance is increased during obesity, which explains, at least in part, why T2D risk is enhanced by obesity. The regulation of glucose homeostasis by insulin is summarized in Fig. 1.

Type 1 diabetes (T1D) is much less common than T2D, accounting for <10 % of cases. It is precipitated by an autoimmune attack on the β-cells that results in an insulin deficient state, although a small number of functioning β-cells may remain [8]. Typically, T1D presents in childhood or young adulthood.

In addition, there are rare inherited monogenic forms of diabetes that usually present in early life, and account for only 1 to 2 % of all diabetes cases. Unlike T2D, where it is believed multiple genes predispose to the disease, monogenic diabetes is caused by mutations in a single gene. Many of these genes encode transcriptional regulators, metabolic enzymes and ion channels that regulate β-cell stimulus-secretion coupling, or they may affect the development of the pancreas. Interestingly, common genetic variants in many of the genes known to cause monogenic diabetes enhance T2D risk; thus, their study may help elucidate the etiology of T2D.

T1D must be treated by insulin injections, due to the lack of β-cells. Therapy for T2D consists initially of dietary control and lifestyle modifications, followed by oral hypoglycemic agents, which may increase insulin secretion (for example, sulfonylureas) or reduce insulin resistance or hepatic glucose output (for example, metformin). If these fail to control hyperglycemia, then insulin is given. Monogenic diabetes is treated in different ways according to the gene involved.

## Why are there no other hormones that can substitute for insulin?

Most control systems, including physiological ones, have built-in redundancy, which ensures that when one system fails another takes over. For example, several hormones can elevate blood glucose. However, only insulin can reduce blood glucose. At first this might seem surprising, but it is worth remembering that too much insulin has far more immediate and devastating effects than too little insulin. If blood glucose falls below 2 mmol/l for as little as 5 minutes, it can cause lethal brain damage. By contrast, it is only when blood glucose is chronically elevated over many weeks and months, due to a sustained lack of insulin, that the complications of diabetes are produced. Thus, insulin is a ‘Goldilocks’ hormone in that both too much and too little are dangerous. But although lack of insulin, and the consequent diabetes, receives much attention, an acute excess of insulin is far more damaging.

Insulin’s other function - its ability to enhance growth - is mirrored by several hormones, such as insulin-like growth factor 1 and 2. It is only the role of insulin in glucose homeostasis that is unique. We therefore speculate that the danger of hypoglycemia is the reason for the unique ability of insulin, acting via a single receptor, to lower blood glucose. In our evolutionary history, when humans battled with inadequate food and unplanned exercise (escaping predators) hypoglycemia was more likely than hyperglycemia. In this situation, a single means of lowering blood glucose is advantageous as there is less chance of inadvertent hypoglycemia. By contrast, the presence of numerous feedback systems to bolster blood sugar is beneficial. Although T2D is an increasing problem in societies today, in evolutionary terms it is of little significance because it generally presents after an individual’s reproductive age. Furthermore, it is only in very recent times that we have been exposed to the plentiful availability of high calorie diets and sedentary lifestyles that drive obesity and T2D.

## How do β-cells avoid inappropriate insulin secretion?

**Fig. 2:**
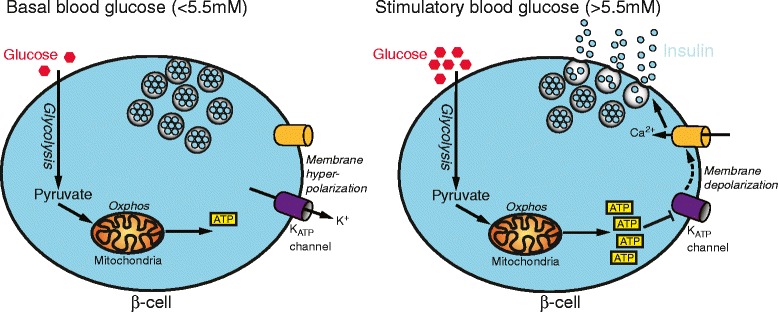
Glucose-stimulated insulin secretion. At basal levels of blood glucose (left-hand panel), the ATP-sensitive K^+^ channels (K_ATP_ channels) in pancreatic β-cells remain open, maintaining membrane hyperpolarization, Ca^2+^ channel closure and inhibiting insulin secretion. A rise in blood glucose (right-hand panel) drives oxidative phosphorylation and ATP production, resulting in the closure of K_ATP_ channels, plasma membrane depolarization, calcium influx and insulin vesicle exocytosis

β-cells have evolved important metabolic features to avoid excessive insulin secretion and hypoglycemia, particularly during exercise. First, insulin secretion is exquisitely sensitive to changes in blood glucose. This is achieved by coupling glucose metabolism with insulin secretion via changes in intracellular ATP levels, β-cell electrical activity and insulin vesicle release. When blood glucose rises, most of the glucose taken up by the β-cell is metabolized via oxidative phosphorylation, thereby elevating intracellular ATP. This closes K_ATP_ channels, so triggering β-cell electrical activity and an influx of calcium (via voltage-gated calcium channels) that, in turn, stimulates insulin release (Fig. 2). Conversely, when blood glucose levels fall, insulin secretion is rapidly switched off due to a reduction in intracellular ATP in β-cells, leading to opening of K_ATP_ channels, membrane hyperpolarization, reduced calcium entry and thereby inhibition of insulin secretion (Fig. 2).

Second, a number of metabolic genes that are widely expressed in other tissues are not expressed in pancreatic β-cells [9-11]. Such ‘disallowed’ genes include those encoding lactate dehydrogenase (LDHA) and the monocarboxylate transporter 1 (MCT1/SLC16A1), which are involved in the metabolism of lactate and pyruvate. This prevents insulin secretion in response to circulating lactate and pyruvate during exercise. Mutations in the *SLC16A1* gene that result in its aberrant expression in β-cells provoke exercise-induced hypoglycemia by enabling pyruvate-induced insulin secretion [12,13]. In early humans, exercise-induced hypoglycemia could be lethal as it would impede escape from a predator; the absence of MCT1 ensures insulin secretion remains switched off during exercise. Similarly, adrenaline inhibits insulin secretion, ensuring blood glucose levels do not drop during exercise or the ‘fight-or-flight’ response.

## What causes the insulin deficiency in type 2 diabetes?

The impaired insulin secretion found in T2D could be due to a decline in the cellular secretory rate (that is, in individual β-cell function), or to a decrease in β-cell mass (the product of β-cell size and number), or both. While there has been much debate about the relative contributions of secretory dysfunction and loss of β-cell mass to impaired insulin secretion in T2D, a consensus view is still lacking. This may, in part, be due to the difficulty in obtaining human islets (especially from T2D donors) of sufficient quality and quantity for functional studies, as islet isolation programs primarily operate to provide islets from healthy donors for transplantation therapy [14]. Furthermore, there are several factors that may vary between human islet preparations, thereby confounding the comparison of control and T2D islet function: donors may have been maintained on different cocktails of drugs prior to death, genetic background and environmental factors may be poorly controlled, and variations in the cold ischemic time to which the islets are exposed during pancreas transport and islet isolation may alter islet gene expression and function [15]. However, when many of the above variables are controlled for, studies with modest sample sizes (n = 5 to 17 cases) have clearly shown that glucose-stimulated insulin secretion (GSIS) is defective in islets from T2D donors, relative to non-diabetic donors [16-18]. In two of these studies, islets from T2D donors responded normally to non-glucose stimuli, suggesting defective GSIS in these cohorts is likely due to impaired glucose-sensing (stimulus-secretion coupling) rather than a loss of insulin content or a constitutive defect in insulin exocytosis [16,18]. Nevertheless, more work is needed, both to increase the numbers of cases studied and to investigate the nature of the defective GSIS response in detail.

Histological studies of β-cell mass are more straightforward because they can be conducted on fixed tissues. Several studies have reported a decrease in β-cell mass in T2D [19-21]. However, an important caveat with these experiments is that β-cells are usually identified by insulin staining. This means that the insulin content must be high enough for it to be detected histologically - β-cells with greatly reduced insulin content will not be counted and β-cell mass thus underestimated. Recent studies indicate that T2D islets contain many β-cells that can be identified as such using electron microscopy by their characteristic ‘poached-egg’ insulin granules, but where the granules are strikingly few and insulin is undetectable by immunostaining [17]. Hyperglycemia produces similar effects in a mouse model of diabetes [22]. Thus, the extent to which β-cell mass is reduced in T2D remains unclear. While there is good evidence that islet insulin staining (aka content) decreases with time, the relative contributions of decreased insulin content, fewer β-cells and impaired stimulus-secretion coupling to reduced insulin secretion in T2D is still uncertain. Notwithstanding this, in the 5 year period following diagnosis, patients with T2D show a 25 % reduction in the mass of insulin-positive cells, relative to non-diabetic controls, whereas in individuals with longstanding T2D (>15 years), β-cell mass is reduced by over 50 % [21]. This progressive loss of β-cell mass during disease progression places an ever-greater secretory burden on the β-cells that remain functional. Their resilience is likely determined by a complex interplay between environmental, genetic and epigenetic factors.

## Do changes in β-cell identity contribute to type 2 diabetes?

It is clear that multiple mechanisms are involved in the development of T2D. However, recent evidence suggests that β-cell identity may not be fixed, and changes in β-cell identity may contribute to defective insulin secretion in T2D.

It is well established that hyperglycemia in mice results in altered expression of β-cell transcription factors and defective insulin secretion, a situation described as β-cell dedifferentiation [23]. Recent elegant studies have shown that deletion of certain transcription factors in mice, such as FOXO1 [24], leads to dedifferentiation of pancreatic β-cells, which lose their insulin content and revert to islet progenitor-like cells. Similarly, expression of the progenitor cell marker Ngn3 has been reported in a mouse model of diabetes [25]. Whether this is the case for human β-cells in T2D is as yet unclear [23]. However, marked changes in β-cell transcription factors are observed in humans with T2D [26] and in non-human primates with diet-induced pre-diabetes [27].

It is well established that the loss of insulin immunostaining seen in many mouse models of diabetes is paralleled by an increase in glucagon immunostaining. These changes appear to be driven by hyperglycemia. In one of these mouse models, lineage tracing revealed that a small number of β-cells start to express glucagon [22]. However, it remains unclear if these β-cells fully convert to α-cells, or if they represent an intermediate cell type that expresses glucagon as well as many β-cell proteins (except for insulin). In contrast, lineage tracing has also shown that both α-cells [28] and δ-cells [29] can convert to fully functioning β-cells, highlighting the potential plasticity of islet cells.

Thus, much of the current research in this area is focused on how to differentiate β-cells from progenitor cells or other islet cell types. Interestingly, the effects of hyperglycemia on β-cell dedifferentiation, loss of insulin content and glucagon expression can be reversed by tight control of blood glucose [22,25].

## Is obesity a driver of β-cell failure?

The current global epidemics of obesity and T2D show remarkably similar trends and geographic distribution, and there is good evidence that the risk of T2D is increased by obesity [30]. However, obesity appears to exert its effect primarily on insulin resistance rather than on β-cell function, and only a minority of obese individuals will develop T2D [31], while many non-obese individuals will do so [32]. Furthermore, obesity is associated with an enhanced insulin response to glucose in non-diabetic individuals [33,34], and recent histological studies have revealed obesity is associated with a 50 % increase in β-cell mass [35]. Thus, it appears that far from obesity causing β-cell failure, the inherent ability for β-cell function to adapt to obesity fails in some individuals, resulting in T2D [31].

## To what extent is type 2 diabetes a genetic disease?

An individual’s risk of developing T2D is determined by a complex interplay between genetic and environmental/lifestyle factors. Genotype clearly plays an important role: prospective studies of monozygotic twins have shown a 76 % concordance rate for T2D, and a 96 % concordance rate for impaired glucose tolerance [36]. Furthermore, a family history of T2D more than doubles an individual’s risk of developing the disease [37]. But at the same time the epidemiological evidence shows a dramatic rise in T2D rates over the past 60 years that clearly cannot be due to genetic change, but is associated with alterations in diet and behavior, including a more sedentary lifestyle and increased consumption of calorie-dense foods [6].

T2D risk may also be influenced by epigenetic changes, which are heritable alterations affecting cell function which do not involve changes in the DNA sequence. These are largely determined by environmental factors, such as parental nutrition. Recent evidence suggests that β-cells from T2D patients show altered DNA methylation (a common epigenetic mark) with changes in gene expression profiles [38]. Rodent studies have shown that suboptimal maternal or paternal nutrition can influence chromatin modifications and gene expression in β-cells of subsequent offspring, consistent with epigenetic transmission [39,40]. In humans maternal and early-life nutrition is known to influence the risk of T2D in offspring [41,42]. Further studies will be needed to clarify the emerging role of epigenetics in the etiology of T2D.

## Are specific gene variants associated with type 2 diabetes?

This is not a straightforward question to answer.

T2D is a polygenic disease, and current evidence favors the idea that in most individuals, the risk of developing the disease is determined by the combination of at-risk variants at many gene loci, each of which alone confers only a small increase in disease risk [43]. This makes T2D distinct from the much rarer monogenic forms of diabetes, such as maturity onset diabetes of the young (MODY) and neonatal diabetes [44]. It also indicates that T2D is not a single entity, as hyperglycemia may be produced by different combinations of genes in different individuals, which may also result in phenotypic variations.

Currently, the best method for identifying genes contributing to polygenic diseases are genome-wide association studies (GWAS). These are based on the association of common genetic variants - single-nucleotide polymorphisms - with a given phenotype, such as hyperglycemia. To date, more than 70 gene loci have been found to be associated with T2D in large-cohort studies [45], with the majority of these loci being implicated in β-cell function.

One problem with GWAS studies is the size of the cohorts that must be studied (sometimes >100,000 people) to generate sufficient statistical power. Such large cohorts are difficult to phenotype in sufficient depth to reveal the complex physiology underlying T2D. Thus, most studies inevitably rely on relatively simple phenotyping procedures, such as measurement of fasting blood glucose, which do not adequately reveal the underlying etiology. Therefore, efforts to improve disease phenotyping are important. A recent study analyzed the association of 37 hyperglycemia susceptibility loci with three key traits that influence blood glucose: insulin sensitivity, β-cell insulin processing and insulin secretion [46]. This revealed that risk loci were clustered into three distinct groups, each associated with only one of the three phenotypic measurements. This study highlights both the marked physiological heterogeneity underlying glycemic traits, and the need for stratification of diabetes phenotypes to enhance the power of GWAS.

New approaches to studying genotype-phenotype interactions in β-cells are urgently needed. Some progress towards this goal was made in a recent study, which analyzed islets isolated from human donors with differing at-risk genotypes. This painstaking approach revealed the direct influence of a subset of diabetes risk loci on impaired insulin secretion *ex vivo* [18], and provided mechanistic insights into the role of these genetic variants.

## Is a personalized treatment for type 2 diabetes, based on genotype, possible?

In some diseases, such as breast cancer, genotyping is routinely used to predict whether a patient will benefit from a particular drug [47]. Genotyping has also revolutionized therapy in some types of monogenic diabetes [48]. The potential for genotype-specific therapy in T2D is less clear, principally because each gene variant only explains a small degree of disease risk. However, a recent study revealed that in T2D patients carrying an alpha-2A-adrenergic receptor mutation, treatment with a drug targeted to this receptor restored insulin secretion [49]. This raises the tantalizing possibility of mining GWAS data for new drug targets to develop personalized treatments for groups of individuals with specific subtypes of T2D.

## Are other islet cell types involved in the pathogenesis of type 2 diabetes?

It is now well established that T2D is not caused simply by lack of insulin and that impaired glucagon secretion from pancreatic α-cells also plays a pivotal role. Glucagon elevates blood glucose by stimulating gluconeogenesis and glucose output from liver hepatocytes. In T2D there is a marked increase in glucagon secretion at high glucose, which exacerbates the hyperglycemic effects of insulinopenia [50]. There is also too little glucagon secretion at low glucose, which may precipitate fatal hypoglycemia [50].

Glucagon, a long-neglected player in glucose homeostasis and T2D, has recently taken center stage. The spectacular finding that the complete destruction of the β-cells by streptozotocin does not result in hyperglycemia in mice in which the glucagon receptor has been genetically ablated [51], whereas wild-type mice are severely diabetic following β-cell ablation, underscores the importance of glucagon in glucose homeostasis. Because expression of the glucagon receptor in the liver alone is sufficient to produce severe diabetes in glucagon receptor-null mice lacking functional β-cells [51], suppression of glucagon-induced hepatic glucose output would appear to be a good target for T2D therapy.

Metformin, widely used to treat T2D (especially in the obese), has been proposed to lower blood glucose by antagonizing glucagon action [52]. This appears to be mediated via reduced cellular metabolism, which leads to inhibition of adenylyl cyclase and cyclic AMP production, and so lowers hepatic gluconeogenesis. Other strategies for reducing glucagon action include reducing glucagon release from pancreatic α-cells [50] and blocking glucagon stimulation of hepatic glucose output [53]. Indeed, glucagon receptor antagonists improve glycemia in T2D [53], and glucagon-like peptide 1 (GLP1) mimetics and inhibitors of dipeptidyl peptidase 4 (DPP4; the enzyme that inactivates GLP-1) are thought to improve glucose homeostasis, at least in part, by reducing plasma glucagon levels [54]. Reduced hepatic glucose output may also be part of the reason why excellent diabetes control can be rapidly achieved (prior to substantial weight loss) by a very low calorie diet [55]. A better understanding of the mechanisms regulating glucagon secretion and action, both in health and disease, and of how these may be targeted therapeutically in T2D is therefore urgently required.

## Can β-cell dysfunction in type 2 diabetes be reversed?

The UK Prospective Diabetes Study Group (UKPDS) demonstrated that there is an inexorable decline in β-cell function with time, whether with diet-control, insulin or sulfonylurea therapies [1]. A key question is what causes this decline and whether it can be reversed.

Improvement in insulin secretion after intensive insulin treatment has been reported [56,57], and a very low-calorie diet can improve insulin action, β-cell function and glucose homeostasis in T2D patients [55]. Thus, some reversal of impaired β-cell function in T2D appears possible, at least in the short term.

Current pharmacological therapies also improve glycemic control in T2D by increasing insulin secretion. These include the sulfonylurea drugs, which act by closing K_ATP_ channels. Drugs that mimic or amplify the action of gut hormones - known as incretins - also enhance insulin secretion. Glucagon-like peptide-1 (GLP-1), for example, is released from intestinal L-cells in response to the presence of food in the gut, and like other incretins, it potentiates insulin secretion at stimulatory glucose concentrations, but not at low glucose [58]. This makes GLP-1 an attractive therapeutic target, because it enhances insulin secretion only in response to a meal, when it is needed, and not during inter-meal intervals when it could increase the risk of hypoglycemia. Thus, GLP-1 mimetics and DPP4 inhibitors are now widely used. They are highly effective at boosting insulin secretion and maintaining glucose homeostasis in T2D patients [54], and they do not cause the weight gain and hypoglycemia risk associated with insulin and sulfonylurea therapies [59]. However, questions have been raised over the long-term safety of incretin-based therapies: in particular, an off-target effect on ductal cells of the exocrine pancreas may increase the incidence of pancreatitis, potentially leading to pancreatic cancer [60,61]. There is debate about whether the potential risk is justified by the profound benefits of incretin-based medications.

Recent studies have revealed that gastric bypass surgery rapidly restores glucose homeostasis in T2D patients, prior to the substantial weight loss associated with this procedure [62]. One explanation for this remarkable finding is that surgery results in an increase in GLP1 secretion and an associated rise in insulin release [63]. Therefore, gastric bypass surgery may be a viable surgical therapy for treating T2D.

## Where can we expect progress in our understanding of β-cell failure in type 2 diabetes?

As ever, research on T2D is a vibrant field and much energy is directed towards understanding the changes in β-cell function associated with diabetes. The root cause of defective stimulus-secretion coupling during T2D remains elusive, and more functional studies using islets isolated from humans with T2D are clearly needed. Currently, there is a renewed focus on β-cell dedifferentiation using mouse models, and how this may be prevented or reversed, and on the extent to which other islet cells can be induced to differentiate into β-cells. The role of glucagon in T2D is also receiving considerable attention, and there is an emphasis on efforts towards better stratification of disease phenotypes in genetic studies. Recent therapeutic developments also hint at the possibility of restoring endogenous β-cell function, although the long-term stability, safety and efficacy of these approaches is not yet known. We look forward to the results of all of these studies.
