# Correction to: Q&A: insulin secretion and type 2 diabetes: why do β-cells fail?

**DOI:** 10.1186/s12915-019-0650-8

**Published:** 2019-04-09

**Authors:** James Cantley, Frances M. Ashcroft

**Affiliations:** Department of Physiology, Anatomy & Genetics, University of Oxford, Parks Road, Oxford, OX1 3PT UK

**Correction to: BMC Biol**

**https://doi.org/10.1186/s12915-015-0140-6**

Upon publication of the original article [1], the authors noticed that they had accidently omitted to acknowledge funding from the European Research Council. The Acknowledgements should have included the sentence “We thank the European Research Council for support (grant no. 322620 to FMA).”
